# Supplementary material for: Trends in SARS-CoV-2 infection prevalence during England’s roadmap out of lockdown, January to July 2021
Source: PLoS Comput Biol. 2022 Nov 23;18(11):e1010724. doi: 10.1371/journal.pcbi.1010724 (PMC9728904; doi:10.1371/journal.pcbi.1010724)
Supplement: S1 Fig — Regional estimates of infection prevalence from 6 January to 12 July 2021 estimated using a Bayesian P-spline model fit to all 13 rounds of REACT-1 (only shown for rounds 8–13) assuming a constant second-order random-walk prior (value set from national model fit). All estimates of infection prevalence are shown with a central estimate (solid line) and 50% (dark shaded region) and 95% (light shaded region) credible intervals. Daily weighted estimates of swab positivity (points) are shown with 95% confidence intervals (error bars). Dashed lines show the date of key restriction changes in England. (DOCX) [file pcbi.1010724.s004.docx]

**
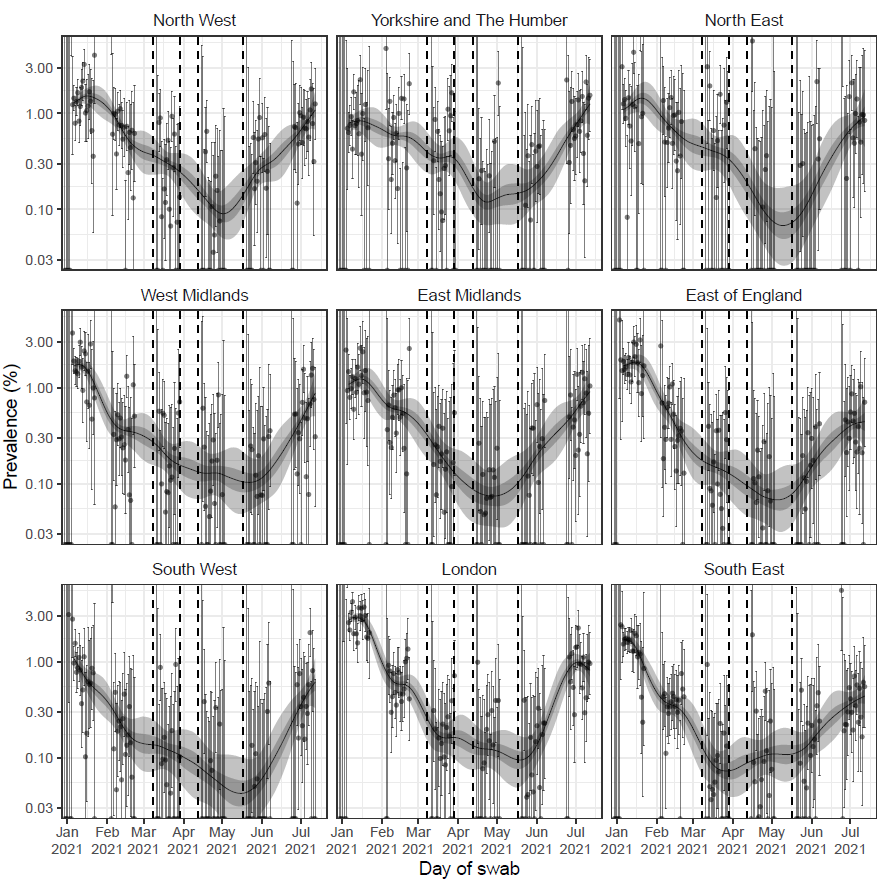
**

**S1 Fig:** Regional estimates of infection prevalence from 6 January to 12 July 2021 estimated using a Bayesian P-spline model fit to all 13 rounds of REACT-1 (only shown for rounds 8-13) assuming a constant second-order random-walk prior (value set from national model fit). All estimates of infection prevalence are shown with a central estimate (solid line) and 50% (dark shaded region) and 95% (light shaded region) credible intervals. Daily weighted estimates of swab positivity (points) are shown with 95% confidence intervals (error bars). Dashed lines show the date of key restriction changes in England.
