# Supplementary material for: Trends in SARS-CoV-2 infection prevalence during England’s roadmap out of lockdown, January to July 2021
Source: PLoS Comput Biol. 2022 Nov 23;18(11):e1010724. doi: 10.1371/journal.pcbi.1010724 (PMC9728904; doi:10.1371/journal.pcbi.1010724)
Supplement: S4 Fig — Rolling two-week average (averaged over prior two weeks) reproduction number as inferred from the Bayesian P-spline models fit to data for each region assuming a gamma distributed generation time with shape parameter = 2.29, and rate parameter = 0.36. Estimates of Reproduction number are shown with a central estimate (solid line) and 50% (dark shaded region) and 95% (light shaded region) credible intervals. The red line shows the probability that R>1 over time. Vertical dashed lines show the dates of key changes in restrictions. Horizontal dashed line shows R = 1 the threshold for epidemic growth. (DOCX) [file pcbi.1010724.s007.docx]

**
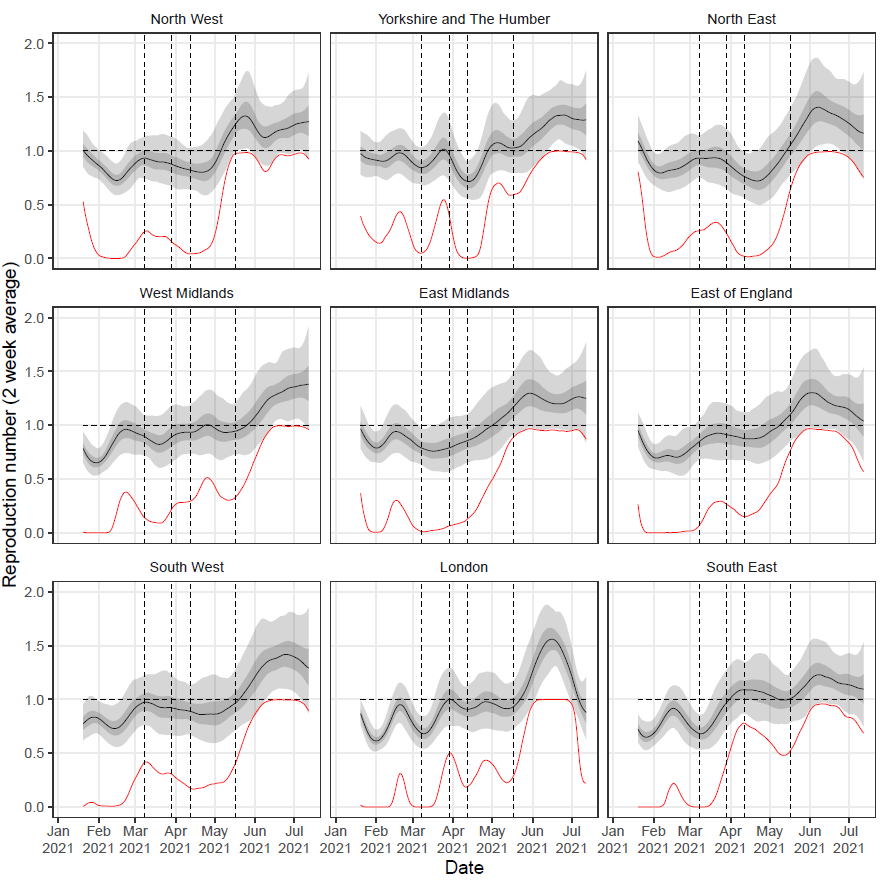
**

**S4 Fig:** Rolling two-week average (averaged over prior two weeks) reproduction number as inferred from the Bayesian P-spline models fit to data for each region assuming a gamma distributed generation time with shape parameter = 2.29, and rate parameter = 0.36. Estimates of Reproduction number are shown with a central estimate (solid line) and 50% (dark shaded region) and 95% (light shaded region) credible intervals. The red line shows the probability that R>1 over time. Vertical dashed lines show the dates of key changes in restrictions. Horizontal dashed line shows R=1 the threshold for epidemic growth.
