# Supplementary material for: Trends in SARS-CoV-2 infection prevalence during England’s roadmap out of lockdown, January to July 2021
Source: PLoS Comput Biol. 2022 Nov 23;18(11):e1010724. doi: 10.1371/journal.pcbi.1010724 (PMC9728904; doi:10.1371/journal.pcbi.1010724)
Supplement: S6 Fig — (A, B) Median estimates for R (points) and 95% credible intervals (error bars) for each period of time between changes in restrictions for the segmented-exponential model fit to subsets of data by region (A) and age-group (B) with the estimates obtained for all data also shown for comparison. (C, D) Median estimates for the multiplicative growth in R (points) and 95% credible intervals (error bars) for each step change in restrictions for the segmented-exponential model fit to subsets of data by region (C) and age-group (D) with the estimates obtained for all data also shown for comparison. In (A,C) Yorkshire is short for Yorkshire and The Humber. (DOCX) [file pcbi.1010724.s009.docx]

**
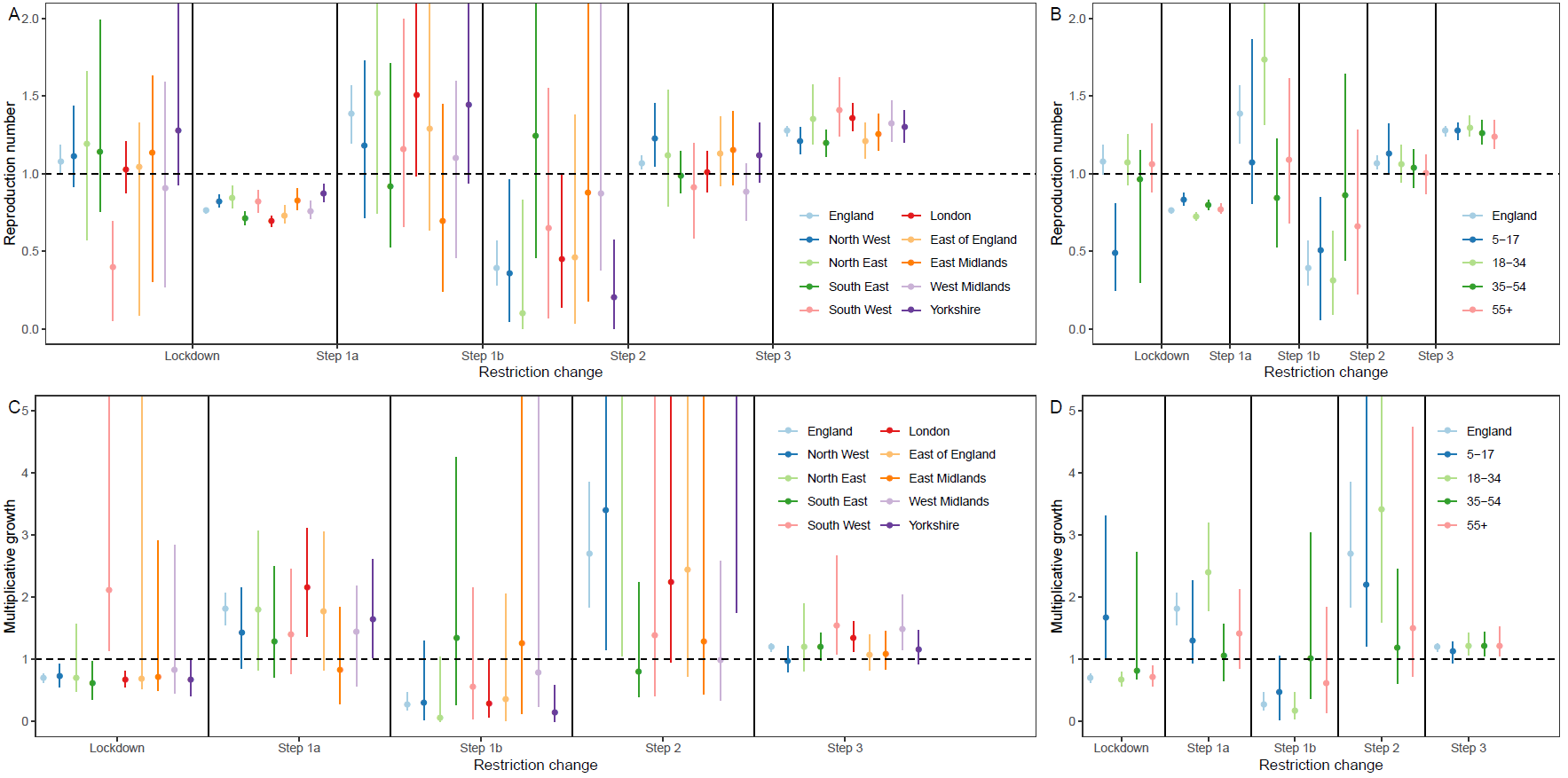
**

**S6 Fig: (A, B)** Median estimates for R (points) and 95% credible intervals (error bars) for each period of time between changes in restrictions for the segmented-exponential model fit to subsets of data by region (A) and age-group (B) with the estimates obtained for all data also shown for comparison. **(C, D)** Median estimates for the multiplicative growth in R (points) and 95% credible intervals (error bars) for each step change in restrictions for the segmented-exponential model fit to subsets of data by region (C) and age-group (D) with the estimates obtained for all data also shown for comparison. In (A,C) Yorkshire is short for Yorkshire and The Humber.
