# Supplementary material for: Trends in SARS-CoV-2 infection prevalence during England’s roadmap out of lockdown, January to July 2021
Source: PLoS Comput Biol. 2022 Nov 23;18(11):e1010724. doi: 10.1371/journal.pcbi.1010724 (PMC9728904; doi:10.1371/journal.pcbi.1010724)
Supplement: Attachment — Submitted filename: Response to reviewers.docx [file pcbi.1010724.s010.docx]

**Reviewer 1**

Authors analyse data from the REACT study in 2021 to show how transmissibility and infection prevalence increased during the re-opening phase. These data are extremely valuable and informative.

Major comments

1. You used a constant generation time distribution throughout, but actually there is good evidence that the generation time for Delta is slightly shorter. How would this impact your results?

We have now included two additional supplementary figures providing Rt estimates nationally and regionally under the assumption of a shorter generation time (estimated for the Delta variant) and updated the results to include the findings. We see similar temporal patterns in Rt under the new generation time, but with very slightly reduced Rt values. We have added a line discussing that our main Rt estimates may be slightly biased upwards during the period towards the end of the study when Delta was the dominant variant.

Minor comments

Abstract line 43 “masked” may not be the best word, it was an intended benefit of vaccination at that time, perhaps a word like “offset” could be considered? On the other hand, Delta had increased intrinsic transmissibility. Maybe “also affected by …”

‘Offset’ has now been used to discuss vaccines and ‘also affected by’ for the Delta variant.

Line 101 — “ensured the results…” I’m not sure you can ensure the results are representative with a low response rate, but this is the best you could do to aim for representativeness.

We have changed this line to ‘ensure the results …. were **more** representative’

Lines 169-170. Did you have information on vaccination status in your participants? This information would have been sufficient to use vaccination information in the regional model? With some general super-parameters for overall VE perhaps.

Unfortunately there was varying quality in the vaccination status data of participants over these rounds. Vaccination status was self-reported and the questionnaire refined over these rounds due to poor quality data in some of the earlier rounds of this study.

On terminology, “school closure” means pretty much everyone stays at home and the school gates are locked. For example a “snow day”. In UK wasn’t it more like “class dismissals” where staff went to the school and arranged online lessons, with most children staying at home but key worker children allowed into school campuses?

I have used the terminology that the government seems to use in the introduction now to make clear before ‘school openings’ that ‘schools were closed to most students’ (instead of just ‘school closures’).

In Figure 3, how to interpret the two-week (prior two week) Rt, does it mean that the Rt on 29 March (transition to step 1b) reflects transmission in the last two weeks of March, or is it intended to be an estimate of the Rt on 29 March ? The latter would be easier to interpret, even if smoothed, because of the way you present the Rt against the policy changes. Specifically, does the bump in Rt on 29 March actually correspond to step 1b or is it a consequence of step 1a two weeks earlier?

It reflects the average rate of transmission over the prior two weeks - so the former. We have added ‘(averaged over prior two weeks)’ into the text to make the interpretation clearer. I agree the latter would be easier to interpret but there would have been far greater uncertainty in a single day's estimate which might have made it hard to interpret with so much noise.

**Reviewer 2**

The authors present a statistical analysis of REACT-1 COVID survey data in the UK, finding an increase in Rt following a relaxation of restrictions. The paper is well written, with clearly explained methods and informative figures. However, I think there are some ways in which the methodology could be strengthened.

1. I was surprised at the narrowness of the uncertainty intervals. This is perhaps most striking in Fig. 5, which has virtually zero uncertainty intervals over some periods, but also e.g. Fig. 3, where the notoriously hard-to-estimate reproduction number also has quite narrow uncertainty bands (such that many of the various wiggles seem to be statistically significant rather than just noise). I have full confidence that the authors have applied the statistical methods correctly, but I am concerned that they have not taken into account other sources of uncertainty.

The uncertainty intervals in figure 5 are very small due to the type of model being fitted. In the segmented exponential model the growth rate is assumed to be constant over a long period of time and it is the average growth rate that is fitted. It is because it is only an average growth rate that the uncertainty is so small. The uncertainty in the time-varying reproduction number is perhaps lower than the reviewer anticipated as it is a two week average estimate and not a daily point estimate of the reproduction number. Uncertainty in the generation time distribution was not taken into account when estimating Rt. However, we have now included an additional sensitivity analysis in which we use a generation time distribution more appropriate for the Delta variant. The results show that the temporal trends in Rt are similar for both generation times used, but now makes it clear that our Rt estimates are a direct conversion of prevalence under the assumption that the generation time distribution is known exactly.

2. For example, the authors note low response rate (11.7%), which could be a significant source of not just uncertainty, but bias (I note also that the swabs were self-administered, which could lead to underreporting). I wonder what Fig. 3 would look like with a different choice of window length (instead of 2 weeks). I wonder what the full ensemble of exponential fits would look like with varying delay lengths, rather than just the single delay shown in Fig. 5. (I also wonder how good the fits were with the segmented-exponential model; these are not shown, but my concern would be that the fewer segments used, the poorer the fit and yet paradoxically the smaller the uncertainty interval.) These methodological limitations would be unlikely to be resolvable within the REACT-1 dataset itself. Thus, the authors are encouraged to cross-validate these results by considering other data sources, such as case counts (which I agree on their own will have more bias than the REACT-1 data), hospital admissions (which will depend on variant), and other data sources (eg wastewater surveillance or seroprevalence). Finally, mobility data could be used to get an independent (and, yes, flawed) estimate of the expected impact of both the lockdowns and the relaxation.

Uncertainty from a low response rate can not be included in any of the methodology as the exact form it takes is unknown. A previous study of REACT-1 has found the self administered swab-tests used in REACT-1 have a high sensitivity (comparable sensitivity values to swab-tests performed by medical professionals) so would not lead to underreporting. The choice of two weeks was chosen as it reflects the period of time in which the generation time distribution goes to approximately zero. Using a three week window instead would thus have little effect on estimates of Rt as the generation time is approximately zero from 14 days to 21 days. We investigated using variable time-delays, but model convergence became a problem and sampling became too biased by the choice of prior distributions used. I agree that the methodological limitations could not be resolved using just REACT-1 data. However, the data being suggested have numerous biases compared to the REACT-1 data and by including them we would instead get biased estimates. It is unfortunate that the sampling rate of the REACT-1 study was not greater and covering all time instead of just discrete rounds.

3. In addition, I am not sure why the authors restricted the study to the dates they did. If REACT-1 was available from May 2020 to March 2022, why not use the whole time series? In particular, I would think the change in Rt from before to after the lockdown would be the single most valuable data point. As it is, this change happens before the analysis period, so its impact cannot be seen.

Some of the similar analysis performed on the other rounds of the REACT-1 study has already been published in other journals. In particular temporal analysis of rounds 1-7 including Rt estimates has been published in Epidemics (<https://www.sciencedirect.com/science/article/pii/S1755436522000482>). I agree that if there had been more data prior to the lockdown it would have been very valuable to include. However, round 7 of REACT-1 ended on 3 December 2020 and so would have added little value to the dataset. Between rounds 7 and round 8 of REACT-1 there was a large peak in infections in England which REACT-1 was not in the field to detect. Including round 7 thus would have not added much but also might have risked biasing estimates away from the large peak that occured between rounds.

4. As it is, the results do not look, to my eye, especially convincing. Squinting at Fig. 3, Rt looks more or less flat from January through mid-May, then increases (but then starts to go down? -- using a longer time series would help). Consider the changes of restrictions as a scalar variable with 0 representing no interactions and 1 representing full interactions. Let's assume values could be estimated for this variable at different points in time (eg, by mobility data). Now consider the value of Rt at, say, 8 post-change as was done with the exponential model. If one then does a regression of these five values (with the restrictions/interactions variable on the x-axis and Rt on the y-axis), it's not clear to my eye that the correlation would be statistically significant. Put even more simply, I would expect the values in Fig. 5A to be monotonically increasing, but they are not.

Though trends in Rt definitely have uncertainty in them, there does appear to be transient changes at the time of restriction changes even if the credible intervals slightly overlap at times. From January to May a flat Rt would be inconsitent with the estimated Rt and its credible intervals. I would not expect the values in Fig. 5A to be monotonically increasing as step 1b to step 2 was at the time of easter holidays. This period saw a clear decrease in social contacts as recorded by the comix surveys and so it was very likely that R would have decreased over this period despite step 1b relaxing some other restrictions. I would also not expect R to be monotonically increasing, as it depends on the effect of vaccination and the Delta variant. I would expect the R estimates in Fig. 5B to be monotonically increasing (except for easter period) which is observed though with great uncertainty for the last two steps (periods of high vaccination and Delta variant).

In summary, while I think this is an interesting study, and while I think the content that is there is relatively strong, I feel a few additional data sources will be required in order to support the authors' claims that "the lockdown was highly effective at reducing risk of infection". It is, if anything, surprising and a little disappointing that a clearer signal cannot be seen in the raw data.

Unfortunately any additional data sources avaialble in the UK, though plentiful, would introduce new and worse sources of bias. The REACT-1 study is the gold standard for surveillance of infection prevalence in England (the ONS study is also good but has restricted access). We have now edited the language around the text to not make as strong a statement about the effectiveness of the lockdown.

**Have the authors made all data and (if applicable) computational code underlying the findings in their manuscript fully available?**

The [PLOS Data policy](https://journals.plos.org/ploscompbiol/s/materials-and-software-sharing) requires authors to make all data and code underlying the findings described in their manuscript fully available without restriction, with rare exception (please refer to the Data Availability Statement in the manuscript PDF file). The data and code should be provided as part of the manuscript or its supporting information, or deposited to a public repository. For example, in addition to summary statistics, the data points behind means, medians and variance measures should be available. If there are restrictions on publicly sharing data or code —e.g. participant privacy or use of data from a third party—those must be specified.

Reviewer #1: Yes

Reviewer #2: **No:** They point to the repository https://github.com/mrc-ide/reactidd, but this does not seem to cover the current paper.

The github repository has now been updated to include a vignette describing the code used to conduct the analysis in the current paper
